# Efficacy of Preoperative Piroxicam, Diclofenac, Paracetamol With Tramadol and Placebo Tablets for Relief of Postoperative Pain After the Removal of Impacted Mandibular Third Molars: A Randomised Controlled Trial

**DOI:** 10.7759/cureus.26839

**Published:** 2022-07-14

**Authors:** Deepankar Shukla, Nitin D Bhola, Rajiv D Bhola, Akshay M Nimje

**Affiliations:** 1 Oral and Maxillofacial Surgery, Sharad Pawar Dental College and Hospital, Wardha, IND; 2 Prosthodontics, Sharad Pawar Dental College and Hospital, Wardha, IND; 3 Research, Narendra Kumar Prasadrao Salve Institute of Medical Sciences and Research Center, Nagpur, IND

**Keywords:** analgesia, nsaids, pain, impacted mandibular third molars, preoperative analgesia

## Abstract

Aim

We aimed to analyze the influence of preoperative piroxicam, diclofenac, paracetamol, tramadol, and placebo tablets as measured in the time required for rescue analgesia for postoperative pain relief after the extraction of impacted mandibular third molar.

Materials & methods

Forty-four patients who needed extraction of impacted mandibular third molar were arbitrarily categorized into four groups namely, piroxicam, diclofenac, paracetamol with tramadol, and placebo. The test medicine was given one hour preoperatively before the surgical removal. The pain was assessed using visual analog scale (VAS) and verbal rating scale (VRS) scores preoperatively and at the third and 24^th^ hours. The time required for escape analgesia was measured.

Results

The mean VAS and VRS scores showed significant differences across the groups after 24 hours. The mean score was lowest for the patients taking piroxicam (1.30+1.95) and highest for patients taking tramadol + paracetamol (4.50+2.59). As far as escape analgesia is concerned piroxicam group was by far superior.

Conclusion

The pain scores and the rescue analgesic requirement suggested that piroxicam analgesic significantly reduced pain; moreover, it is a safe as well as an efficacious substitute to the conventional non-steroidal anti-inflammatory drugs (NSAIDs) for mandibular third molar impactions.

## Introduction

Wisdom tooth impaction is a commonly diagnosed problem that often demands tooth removal. Pain, difficulty in opening the mouth, and edema are frequent consequences following surgical extraction of impacted third molars. This is a result of localized inflammatory mediators, in particular cyclooxygenase (COX) and prostaglandins [1]. Good surgical techniques may help minimize but do not eliminate postoperative pain and inflammation. [2]. 

The degree of pain (mild, moderate, or severe), quality (sharp, throbbing, or dull), duration (transient, intermittent, or constant), and radiation (localized or diffuse) can all change. Despite pain being a largely sensory experience, it has significant cognitive and psychological effects; it is related to or defined by suffering. According to The International Association for the Study of Pain (IASP), pain is "an unpleasant sensory and emotional experience associated with present or potential tissue damage or characterized in terms of such harm" [3]. 

People often link dental treatments with discomfort, which makes them avoid or postpone treatment, which makes it more difficult to manage [4]. The effectiveness of analgesics for acute dental pain is investigated by employing a common model of extraction of an affected third molar [5]. Pain control protocols after third molar surgery are critical for providing optimal dental care. Although at present, the arsenal of local anesthetic and analgesic medicines is often sufficient, the inability to effectively manage early postoperative pain is upsetting for both patients and professionals [6].

Postoperative analgesia can be delivered through the application of local anesthesia or the administration of non-steroidal anti-inflammatory drugs (NSAIDs), opioids, or a combination of the two. Preoperative use of NSAIDs possibly is more beneficial than postoperative use [7]; similarly, opioids are also more efficacious if employed prior to the surgery [8-10]. The purpose of administering preoperative analgesia is to avoid or lessen ensuing pain. Pre-emptive analgesia has the effect of preventing or reducing the formation of "memory" of painful stimuli in the central nervous system. The possibility of enhancing postoperative pain management is of clinical importance [11]. The metabolites of leukotrienes, arachidonic acid, and prostaglandins are widely recognized to be involved in the process of inflammation, acting as mediators and regulators of causing vasodilation and enhanced capillary permeability that promotes the movement of macrophages towards the location of the inflammation. NSAIDs can be administered to treat pain, trismus, as well as edema because they block the enzyme cyclooxygenase and thereby limit the formation of prostaglandins prostaglandin E2 (PGE2) and prostaglandin I2 (PGI2) [12,13].

NSAIDs are beneficial for treating postoperative pain. The most probable method of action is inhibition of prostaglandin production [14]. Paracetamol (PCM) is not a narcotic painkiller and has been studied for dental pain following operative procedures. Its action mechanism is not known. Recent evidence from animal-based and human studies agrees with the concept due to the stimulation of the descending serotonergic system, PCM exerts a central analgesic effect, but its principal site of action is the suppression of prostaglandin synthesis. According to biochemical investigations, cyclooxygenase-3 (COX-3) activity that is preferentially sensitive to PCM may be an alternate site of action [15,16]. Piroxicam is a non-selective cyclooxygenase inhibitor that belongs to a broad class of analgesics and NSAIDs. Piroxicam is well absorbed 30 minutes after oral administration. It is particularly beneficial for the treatment of many forms of pain and inflammatory processes when given at a suggested dose of 20 mg once a day or 10 mg twice a day [17].

Diclofenac is an NSAID that comes as potassium salt tablets for oral administration as well as sodium salt extended-release and delayed-release tablets. Diclofenac has shown an advantage in rodent acute inflammatory models caused by kaolin, carrageen, or adjuvant arthritis in pharmacologic investigations, in part due to suppression of the enzymatic transition of arachidonic acid into prostaglandin at extremely low concentrations [18]. Diclofenac seems to have a strong inhibition effect on cyclooxygenase 1 and 2 sites [19]. Diclofenac is swiftly plus effectively absorbed from the digestive tract, according to pharmacokinetic studies. The area under the plasma peak concentrations and plasma concentration-time curve (AUC) is directly proportional to dosages ranging from 25 to 100 mg, irrespective of administration mode, and there is no build-up with recurring treatments [20]. Tramadol is made up of two isomers that have different activity spectra [21]. It stimulates both opioid and non-opioid (descending monoaminergic) pain-inhibition systems [22]. Tramadol's non-opioid component works by blocking norepinephrine and 5-hydroxytryptamine re-uptake and, presumably, displacing stored 5-hydroxytryptamine from nerve terminals through a2-agonistic and serotoninergic effects [23].

The objective of this randomized, placebo-controlled, double-blind study is to see if piroxicam, diclofenac, PCM with tramadol, and placebo tablets could help relieve postoperative pain after removal of the affected mandibular third molar.

## Materials and methods

This randomized controlled trial was carried out at Saveetha Dental College and Hospitals in Chennai, in the Department of oral and maxillofacial surgery (OMFS). In 44 patients, the researchers compared the preoperative pain relief effectiveness of oral piroxicam, diclofenac, PCM, tramadol, and placebo on pain management following surgical removal of affected third molars. The protocol was authorized by Institutional Ethical Committee, and the study followed the ethical criteria for human experimentation outlined in the Declaration of Helsinki (2000). Before taking part in the study, all patients supplied written informed consent. The study included patients in age between 18 to 45 years with impacted mandibular third molar.

Patients who had used analgesics or sedatives before surgery, pregnant or lactating women, and those with systemic disorders were all excluded from the study. Previously reported observations were utilized to calculate the sample size. A sample size of 44 participants was determined by a power calculation of 90%. The investigator recorded the duration of operation as well as the impaction score for each tooth (based on Pell & Gregory's classification as explained by Pederson) at the time of surgery. Treatment regimen allocation was randomized using block randomization with a block size of eight. The patient was blinded with regard to the drug being given. 

Patients were placed in four different groups: patients in group I got 20mg of piroxicam orally an hour prior to the extraction of impacted mandibular third molars, while patients in group II received 50mg of diclofenac sodium orally an hour prior to the extraction of impacted mandibular third molars. Patients in group III got 37.5mg tramadol + 325mg PCM orally an hour prior to the extraction of impacted mandibular third molars, while patients in group IV received placebo (capsule becosule) orally an hour prior to the extraction of impacted mandibular third molars.

Surgical procedure

Extraction of the affected third molar surgically was performed on all the patients under local anesthesia (lignocaine 2%, with 1:200000 epinephrine). The inferior alveolar nerve (IAN), lingual nerve, and long buccal nerve were blocked. Ward's incision was made from the distobuccal aspect of the second molar. Extended anteriorly and inferiorly, continued along the buccal aspect of impacted third molar and extended distally and laterally. A mucoperiosteal flap was reflected to expose the tooth. Bone was removed with bur under continuous irrigation of sterile isotonic saline solution to reduce the heat generated, followed by splitting and elevation of the impacted tooth. The bony margins were filed and smoothened, and the gingival margins were trimmed. The wound was irrigated with a sterile isotonic solution. The flap was repositioned, and the wound was sutured with non-resorbable black silk suture material. A pressure pack was given to attain hemostasis. Postoperatively, for all patients, amoxicillin 500mg thrice daily was given. The sutures were removed for all patients on the seventh postoperative day.

Rescue medication

As a rescue medication, aceclofenac (100mg) + PCM (325mg) combination was given to patients who did not attain sufficient pain relief. Patients who had consumed rescue medication before three hours were excluded from the final analysis.

Efficacy assessment

Visual Analogue Scale (VAS)

Patients had to use the VAS scale with readings from 0 to 10 with a gradation of 1. The pain intensity was recorded one hour before surgery, immediately before taking a pre-emptive analgesic, then in the third and 24th hour after the surgery. Anchor points were 0, which indicated no pain, and 10, which indicated severe pain. 

Verbal Rating Scale (VRS)

Patients were asked to use a VRS scale with readings as a) no pain, b) mild, c) moderate, and d) severe pain.

Time to remedication

The time to remedication is the time between the termination of surgery and the patient's need for rescue medication. The time that a participant received rescue treatment (aceclofenac + PCM) was recorded.

Descriptive and inferential statistics were employed to execute the statistical analysis, along with the Kruskal-Wallis test, Mann-Whitney test, and Chi-square test. The software used in the study was SPSS version 27.0 (IBM Inc., Armonk, New York), and the significance level was analyzed as p<0.05.

## Results

All of the patients followed the research protocol, endured the surgical procedures well, and returned for follow-up appointments. Forty-four patients who met our eligibility criteria agreed to take part in this study and underwent surgical removal of affected third molars. Out of these 44 patients, one patient was withdrawn because of an adverse drug reaction, and two patients had to be excluded from the analysis because of intake of rescue medication before the third-hour evaluation of pain.

Demographics

Out of 44 patients in this study, 10 patients in the diclofenac group had eight males and two females. Out of 10 patients who were in the piroxicam group, seven were males and three were females. There was an equal distribution of males and females in PCM with the tramadol group. There were five males and six females in the placebo group of 11 patients. There was no substantial distinction in terms of patient distribution based on impaction difficulty (Table 1).

**Table 1:** Kruskal-Wallis test to compare the mean values between groups

	Group	N	Mean	Std. Dev	Min	Max	P-value
Difficulty level	Diclofenac	10	5	0.943	4	6	0.32
	Piroxicam	10	4.7	1.418	3	7	
	Placebo	11	4.09	1.136	3	6	
	Tramadol + paracetamol	10	4.7	1.252	3	6	
	Total	41	4.61	1.202	3	7	

Pain scores

Table 2 depicts the mean VAS scores between groups over time. The mean VAS score did not show any substantial difference between the groups before the surgical procedure and three hours postoperatively (p>0.05).

**Table 2:** Kruskal-Wallis Test to compare the mean VAS scores between groups over time VAS - visual analogue scale; ESC ANG - escape analgesia

VAS	Drug	N	Mean	Std. Dev	Min	Max	P-value
Before	Diclofenac	10	1.7	1.83	0	6	0.763
	Piroxicam	10	2.6	2.37	0	8	
	Placebo	11	2	2.05	0	6	
	Tramadol + paracetamol	10	1.7	2.62	0	5	
	Total	41	2	1.92	0	8	
After 3 hours	Diclofenac	10	3.9	3.18	1	8	0.435
	Piroxicam	10	2.4	1.51	0	5	
	Placebo	11	4.09	2.26	1	8	
	Tramadol + paracetamol	10	2.7	2.31	0	8	
	Total	41	3.29	2.41	0	8	
After 24 hours	Diclofenac	10	3.4	3.13	0	8	0.02
	Piroxicam	10	1.3	1.95	0	6	
	Placebo	11	2.36	1.12	1	5	
	Tramadol + paracetamol	10	4.5	2.59	1	8	
	Total	41	2.88	2.51	0	8	
ESC ANG (hours)	Diclofenac	5	8	8.97	3	24	0.001
	Piroxicam	1	5	-	5	5	
	Placebo	11	2	0	2	2	
	Tramadol + paracetamol	4	4.88	1.44	3	6	
	Total	21	4.12	4.77	2	24	

The mean VAS score showed a significant difference across the groups after 24 hours. The mean score was lowest for the patients taking piroxicam (1.30+1.95) and highest for patients taking tramadol + PCM (4.50+2.59). The difference across the groups at 24 hours was statistically significant (p<0.05).

Table 3 depicts Mann-Whitney test to compare the pairwise mean and demonstrates no notable difference between diclofenac vs. piroxicam, diclofenac vs. placebo, diclofenac vs. tramadol + PCM (p>0.05) after 24 hours but there is a significant difference between Piroxicam vs. placebo, piroxicam vs. tramadol + PCM, placebo vs. tramadol + PCM (p<0.05).

**Table 3:** Mann-Whitney test to compare the pairwise means VAS - visual analogue scale; ESC ANG - escape analgesia

	Group	P-value
VAS after 24Hrs	Diclofenac vs. piroxicam	0.074
	Diclofenac vs. placebo	0.717
	Diclofenac vs. tramadol + paracetamol	0.378
	Piroxicam vs. placebo	0.036
	Piroxicam vs. tramadol + paracetamol	0.005
	Placebo vs. tramadol + paracetamol	0.049
ESC ANG	Diclofenac vs. piroxicam	0.546
	Diclofenac vs. placebo	<0.001
	Diclofenac vs. tramadol + paracetamol	0.71
	Piroxicam vs. placebo	0.001
	Piroxicam vs. tramadol + paracetamol	0.999
	Placebo vs. tramadol + paracetamol	0.001

Table 4 shows the proportion of patients in each group that require rescue analgesia. Five patients in the diclofenac group took rescue analgesia, one in the piroxicam group, and four in the PCM with tramadol group.

**Table 4:** Chi-square test to compare the proportion of patients who required the additional dose of analgesia in each group ESC ANG - escape analgesia * indicates a p-value of less than 0.05

Group	ESC ANG needed						P-value
	No		Yes		Total		
	N	%	N	%	N	%	
Diclofenac	5	50	5	50	10	100	0.001*
Piroxicam	9	90	1	10	11	100	
Placebo	0	0	11	100	11	100	
Tramadol + paracetamol	6	60	4	40	11	100	
Total	20	48.8	21	51.2	43	100	

Table 5 depicts Chi-test to compare the proportions of VRS score between groups, which shows no significant difference between groups before surgery and three hours after surgery (p>0.05). However, there was a considerable difference between the groups after 24 hours (p< 0.05).

**Table 5:** Chi-square test to compare the proportions of VRS score between groups VRS - verbal rating scale * indicates a p-value of less than 0.05

Verbal response scale		Drug Group								Total		P-value*
		Diclofenac		Piroxicam		Placebo		Tramadol+ paracetamol				
		N	%	N	%	N	%	N	%	N	%	
Before surgery	No	2	20	1	10	4	36.4	2	20	9	22	0.962
	Mild	7	70	7	70	6	54.5	7	70	27	65.9	
	Moderate	1	10	1	10	1	9.1	1	10	4	9.8	
	Severe	0	0	1	10	0	0	0	0	1	2.4	
	Total	10	100	10	100	11	100	10	100	41	100	
After 3 hours	No	0	0	1	10	0	0	2	20	3	7.3	0.389
	Mild	6	60	7	70	6	54.5	7	70	26	63.4	
	Moderate	2	20	2	20	4	36.4	0	0	8	19.5	
	Severe	2	20	0	0	1	9.1	1	10	4	9.8	
	Total	10	100	10	100	11	100	10	100	41	100	
After 24 hours	No	2	20	5	50	0	0	0	0	7	17.1	0.018
	Mild	5	50	4	40	10	90.9	5	50	24	58.5	
	Moderate	1	10	1	10	1	9.1	3	30	6	14.6	
	Severe	2	20	0	0	0	0	2	20	4	9.8	
	Total	10	100	10	100	11	100	10	100	41	100	

## Discussion

Extraction of the affected mandibular third molars surgically is one of the most common dentoalveolar interventions in oral and maxillofacial surgery, which is linked with variable degrees of postoperative discomfort. The most common postoperative complaints are pain, trismus, and edema; hence it may affect the quality of life of a patient in the days post-surgery [24].

Acute tissue injury caused by surgical operations results in activation of peripheral nociceptors, which has an impact on the central nervous system. The resulting primary and secondary hyperalgesia best exemplifies the extensive influence of local site injuries on the nervous system [25]. Primary hyperalgesia, characterized by an enhanced sensitivity toward thermal and mechanical stimuli [26], occurs due to the sensitization of peripheral cutaneous receptors by inflammatory mediators such as prostaglandins [27]. Secondary hyperalgesia is due to an increase in sensitivity towards mechanical stimulus in areas surrounding the site of damage [3]. Secondary hyperalgesia is caused by central sensitization of trigeminal nucleus neurons along with the dorsal horn and according to new data, supra-spinal structures, including the rostral ventral medulla, play a significant role in the establishment and maintenance of sympathetic activation and secondary hyperalgesia [28]. 

Recent literature suggests that prostaglandins and cyclooxygenases, in addition to their roles in the peripheral nervous system, also have crucial roles in the central nervous system [29]. As a result, primary hyperalgesia mainly involves the peripheral processes, which can lead rise to secondary hyperalgesia primarily using central mechanisms and cause pain postoperatively. In most circumstances, because postoperative pain is a predicted event, actions should be undertaken to reduce patient discomfort associated with surgical operations.

Prostaglandins are crucial molecules involved in peripheral nociceptors sensitization, which leads to primary and secondary hyperalgesia. Prostaglandins are produced by activating phospholipase A2 in damaged tissue, which results in the creation of arachidonic acid from membrane phospholipids. After that, cyclooxygenase (COX)-dependent mechanisms convert arachidonic acid to different prostaglandins. Prostaglandin D2 (PGD2), PGE2, thromboxane A2 (TxA2), PGI2 (prostacyclin), and prostaglandin F2 (PGF2) alpha are the five major prostanoids in the prostaglandin family. These prostaglandins then have an effect on the body by attaching to certain G-linked receptor proteins. The prostanoid PGE2 is especially important in acute inflammation, and its levels are linked to the degree of pain following wisdom teeth extraction [30]. 

As prostaglandins are not present in large quantities in normal tissues to be active and must first be generated, they aren't involved in the first reaction to pain that leads to tissue damage [31]. After tissue injury, prostaglandins are rapidly produced and emerge in high quantities one hour later. COX-1 is found in a variety of organs and is involved in gastric mucosa protection, platelet aggregation, and renal blood flow control [32]. COX-1 is sometimes referred to as the constitutive COX enzyme because it is found in normal tissues. COX-2 is present in many normal tissues, including the glomerulus, kidney, spinal cord, brainstem, and brain; nevertheless, COX-2 expression is increased in pathological circumstances like chronic inflammatory diseases; hence it is also known as the inducible COX enzyme [33].

The idea of using NSAIDs to alleviate postoperative pain, either as a single therapy or combined with other medications, is getting popular as more is revealed about their mechanisms of action [34]. NSAIDs inhibit prostaglandin formation by COX-1 and COX-2 pathways - inhibition of COX-1 could be implicated with the formation of common NSAID side effects, while blockage of COX-2 leads to analgesia [35]. There are several methods for dealing with pain. The revelation that central sensitization of intrinsic dorsal horn neurons in animal pain models might be reduced if drugs were administered before the injury occurred highlighted an important potential development in the treatment of pain following surgeries [3,36]. The reduction in changes in these neurons found when the drugs are administered prior to the damage gives rise to the idea of pre-emptive analgesia [37]. According to scientific research, an analgesic given pre-surgery seems to have a better outcome than the same drug given post-surgery. The research indicates that there was no significant variation in the third hour after surgery, although the piroxicam group was superior at the 24th hour.

The aim of this study is to compare the effect of preoperative analgesia in the four groups as measured in the time required for rescue analgesia for the relief of postoperative pain. Medication with the aceclofenac-PCM combination was allowed to the patient on the occurrence of pain postoperatively before the scheduled dosage of the test drug. The piroxicam group consumed less rescue analgesia than the other groups. In a study by Ong et al., prophylactic intravenous ketorolac 30 mg was shown to be more beneficial than tramadol 50 mg in preventing postoperative tooth discomfort [38]. Another study found that individuals who received preoperative meloxicam 15 mg reduced pain intensity and overall analgesic intake compared to 50 mg tramadol preoperatively [39].

Tramadol is an excellent postoperative painkiller; however, the considerable adverse effects of vomiting and nausea limit its widespread use [40]. Since 37.5mg of tramadol was utilized in our trial, no negative effects were seen. Rousan et al. demonstrated that preoperative diclofenac sodium medication provided effective alleviation from mild to moderate pain following wisdom tooth surgery as compared to postoperative dosing [41]. Tuzuner et al. investigated the postoperative pain-relieving effects of prophylactic intravenous PCM (perfalgan) and in individuals undergoing impacted wisdom teeth surgery, intramuscular diclofenac sodium and intravenous lornoxicam were used. According to the findings, preoperative diclofenac, lornoxicam, and intravenous PCM effectively reduced postoperative flurbiprofen intake and pain severity [42].

This is the first of its kind of study to compare piroxicam, diclofenac, and PCM to tramadol before surgery. Piroxicam, a non-selective cyclooxygenase inhibitor, is one of the NSAIDs which is beneficial for the treatment of various types of pain and inflammatory processes when taken at a suggested dose of 20 mg once a day or 10 mg twice a day [17]. Kohli et al. compared pain medication with 400 mg of ibuprofen or placebo, attempting to incorporate 20 mg of piroxicam resulted in reporting much less pain. Piroxicam has a significant half-life and consequently a more prominent effect than diclofenac and PCM combined with tramadol. It is possible that using piroxicam as a preoperative analgesic result in greater postoperative pain relief [43].

## Conclusions

Pain control is the most critical aspect to be considered following impacted teeth surgical removal for effective perioperative pain management. In this study, piroxicam 20mg given one hour preoperatively significantly reduced rescue analgesia requirement. This study adds to the growing knowledge of the use of piroxicam as preoperative analgesia in perioperative pain management during third molar surgery. Piroxicam, a non-selective cyclooxygenase inhibitor, is one of the NSAIDs which is beneficial for the treatment of various types of pain and inflammatory processes when taken at a suggested dose of 20 mg once a day or in a divided dose.
